# A postmenopausal women presenting with atypical symptoms and cervical cancer: a case report

**DOI:** 10.1186/1757-1626-1-401

**Published:** 2008-12-16

**Authors:** Hooman Soleymani Majd, Sean Watermeyer, Essam El Hamamy, Lamiese Ismail

**Affiliations:** 1 Milton Keynes General Hospital, Department of Obstetrics & Gynaecology, Standing Way, Eaglestone, Milton Keynes, MK6 5LD, UK; 2 Royal Gwent Hospital, Department of Obstetrics & Gynaecology, Cardiff Road, Newport, NP20 2UB, UK; 3 Royal Gwent Hospital, Department of Obstetrics & Gynaecology, Cardiff Road, Newport, NP20 2UB, UK; 4 Homerton University Hospital, Department of Obstetrics & Gynaecology, Homerton Row, London, E9 6SR, UK

## Abstract

**Background:**

Globally cervical cancer kills millions of women every year. There is a wealth of evidence suggesting that cervical screening is one of the best defences against the development of cervical cancer. Lives could be saved if medical practitioners make a point of routinely enquiring about the date and result of the patient's last cervical smear test and if they repeatedly emphasize the importance of attendance for cervical smear tests, especially in post-menopausal women.

**Case presentation:**

A 66 year old caucasian woman presented with symptoms of a lower respiratory tract infection, weight loss, anorexia and night sweats. There was no history of post menopausal bleeding. She was admitted for intravenous antibiotics. A few days later she developed vomiting, abdominal pain and a brown vaginal discharge.

She then had a CT scan which showed a pyometra extending to the umbilicus, with an intrauterine contraceptive device noted inside. After re-taking the history, it emerged that a Lippes loop was inserted 25 years previously. The patient was not given relevant information at the time and then unfortunately was lost to follow up.

The pyometra was drained and the coil removed. However, at operation cervical cancer was suspected and biopsies taken. The patient's sepsis improved after pyometra drainage but histology subsequently confirmed stage 1B squamous cell cervical carcinoma. She was referred for a radical hysterectomy.

**Conclusion:**

Every consultation is an opportunity for health education and promotion. Patients need to be encouraged to utilize cervical screening programmes. It is also important to remember that cervical cancer can present with non-specific symptoms, thus the onus is on all doctors to take a good history and perform a thorough examination. Failing to do so may delay making the right diagnosis, with associated morbidity and mortality.

## Background

Cervical screening is one of the best defences against the development of cervical cancer, which is the sixth commonest cause of cancer deaths in the UK. Clinicians should make a point of routinely enquiring about the date and result of the patient's last cervical smear test.

It is vitally important for medical practitioners to repeatedly emphasize the importance of attendance for cervical smear tests, especially in post-menopausal women. As many of them may never have been screened. This could provide an important opportunity for health education, as many older women are still under the misconception that cervical cancer is a disease that only affects young, promiscuous women.

## Case presentation

A 66 year old Caucasian, post-menopausal woman was admitted to hospital under the care of the general physicians. She had a three months history of cough and productive green sputum, weight loss, poor appetite and frequent night sweats. Interestingly there was no history of vaginal bleeding.

Clinical examination revealed she was febrile. On auscultation of the chest, crepitations were audible. The abdomen was soft but tender on palpation of the lower abdomen.

Initial investigations included an elevated CRP (279.8) and white cell count (25.4). She was anaemic with a haemoglobin of 8.2 g/dL. Chest X-Ray showed basal atelectasis but nil else.

The patient was transfused with two units of blood and broad spectrum intravenous antibiotics were commenced. A few days later the patient developed vomiting, abdominal pain and was noted to have a brown vaginal discharge. In view of her continuing poor general condition, the lower abdominal tenderness and discharge, imaging of the abdomen and pelvis was arranged.

**Figure 1:**
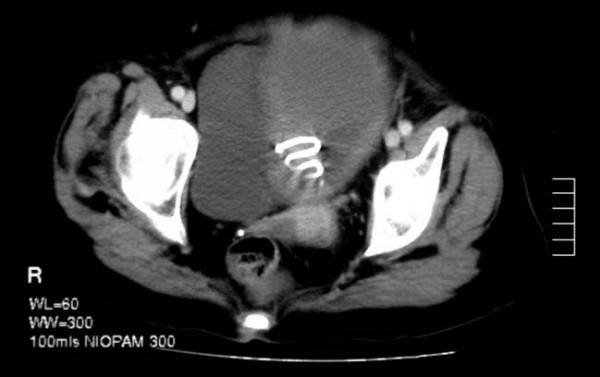
CT scan showing large pyometra with intra-uterine contraceptive device in-situ.

A CT scan revealed a huge pyometra extending up to the umbilicus and an intrauterine contraceptive device was noted to be in situ (Figure 1). No lymphadenopathy was evident and the remainder of the abdomen was normal. The patient's care was then transferred to the gynaecology team.

After taking a thorough gynaecological history, it emerged that the patient had a Lippes loop inserted 25 years previously. She was not counselled regarding the importance of re-attending to have the coil checked or told that she would need to have it removed at some point. In addition to this, the patient was not aware that she needed to have regular cervical smear tests and was then unfortunately lost to follow up. Closer examination revealed a tender 24 weeks size uterus.

She was taken to theatre, where 1600 ml of pus was drained from the uterus and the coil removed. However, at operation the cervix was noted to be suspicious. Biopsies were taken and further examination under anaesthesia clinically confirmed cervical cancer.

After drainage of the pyometra and administration of appropriate antibiotics, the patient made a good recovery from her septic state. Histology later confirmed a stage 1b poorly differentiated squamous cell carcinoma of the cervix. She was referred to the oncologists and is due to have a radical hysterectomy.

## Discussion

Interestingly, the literature suggests that there is a correlation between intra-uterine contraceptive devices and a lowering of the risk of squamous cell carcinoma of the cervix [1]. The rationale for this is that if the cervix is viewed during insertion, removal or checking of the intra-uterine contraceptive device then opportunities for cervical screening would be improved.

Thus women with intra-uterine contraceptive devices need to be informed (verbally and preferably in writing) of appropriate follow up arrangements with regards to their contraception. As non attendance by these patient's could lead to significant morbidity as demonstrated in this case.

## Conclusion

This case also reflects the fact that cervical cancer can present with rather non-specific symptoms. Thus the onus is on all doctors, of all grades to take a good history and perform a thorough physical examination. Failing to do so may delay making the right diagnosis and had there been more of a delay, the pyometra may have ruptured resulting in generalized peritonitis, which could have proved fatal[2].

Every consultation is an opportunity for health education and promotion. We know that cervical screening is a good tool to detect early changes that could ultimately lead to cancer, now all we have to do is encourage and remind patients that they should take it up.

## Abbreviations

CRP: C reactive protein; CT: computerized tomography.

## Consent

Written informed consent was obtained from the patient for publication of this case report and accompanying images. A copy of the written consent is available for review by the Editor-in-Chief of this journal.

## Competing interests

The authors declare that they have no competing interests.

## Authors' contributions

SW and EE were the consultants looking after and managing the patient in question, they also performed her surgery. HS was another member of the clinical team who looked after the patient post operatively and obtained her consent for publication. All three were involved in the writing of the case report as well. LI helped HS analyze the patient data. They conducted literature searches and wrote the case report, preparing it for publication. All authors read and approved the final manuscript.
